# Validation of a New Multicistronic Plasmid for the Efficient and Stable Expression of Transgenes in Microalgae

**DOI:** 10.3390/ijms21030718

**Published:** 2020-01-22

**Authors:** Ana Molina-Márquez, Marta Vila, Rocío Rengel, Emilio Fernández, Federico García-Maroto, Javier Vigara, Rosa León

**Affiliations:** 1 Laboratory of Biochemistry. Faculty of Experimental Sciences. Marine International Campus of Excellence and RENSMA. University of Huelva, 21071 Huelva, Spain; marta.vila@dqcm.uhu.es (M.V.); ro.rd89@gmail.com (R.R.); jvigara@uhu.es (J.V.); rleon@uhu.es (R.L.); 2 Department of Biochemistry and Molecular Biology. University of Córdoba, 14071 Córdoba, Spain; bb1feree@uco.es; 3 Laboratory of Biotechnology of Natural Products, Agro-feed International Excellence campus, University of Almería, 04071 Almería, Spain; fgmaroto@ual.es

**Keywords:** 2A, microalgae transformation, paromomycin, multicistronic transcript

## Abstract

Low stability of transgenes and high variability of their expression levels among the obtained transformants are still pending challenges in the nuclear genetic transformation of microalgae. We have generated a new multicistronic microalgal expression plasmid, called Phyco69, to make easier the large phenotypic screening usually necessary for the selection of high-expression stable clones. This plasmid contains a polylinker region (PLK) where any gene of interest (*GOI*) can be inserted and get linked, through a short viral self-cleaving peptide to the amino terminus of the aminoglycoside 3′-phosphotransferase (APHVIII) from *Streptomyces rimosus*, which confers resistance to the antibiotic paromomycin. The plasmid has been validated by expressing a second antibiotic resistance marker, the *ShBLE* gene, which confers resistance to phleomycin. It has been shown, by RT-PCR and by phenotypic studies, that the fusion of the *GOI* to the selective marker gene *APHVIII* provides a simple method to screen and select the transformants with the highest level of expression of both the *APHVIII* gene and the *GOI* among the obtained transformants. Immunodetection studies have shown that the multicistronic transcript generated from Phyco69 is correctly processed, producing independent gene products from a common promoter.

## 1. Introduction

Microalgae are a heterogeneous group of photosynthetic microorganisms highly attractive for the production of many different interesting metabolites. However, the high cost of microalgal culture systems and the low productivity of many of these compounds have hampered their commercial applications. Currently, only high added value compounds, such as pharmaceuticals, cosmetics, or nutraceuticals, are produced from microalgae at industrial scale [1]. In other fields, competition with synthetic products or with those obtained from other sources makes the production of microalgal-based compounds non-viable from the economical point of view [2,3,4,5,6]. As a consequence, more attention is being paid to the genetic engineering of microalgae as a potential tool to achieve economically feasible production of bulk materials and to enhance the productivity of the high added value ones [7,8,9]. Although in recent years, an increasing number of microalgal species have been successfully transformed and important goals have been accomplished, the genetic transformation of microalgae has still important pending challenges. Both, the low stability of the transgenes and the high variability of their expression levels among the obtained transformants, make necessary large screenings to select high-expression stable clones [10,11,12]. In fact, low nuclear expression of transgenes in microalgae has hampered the efficient and stable engineering of metabolic pathways and has limited the use of these microorganisms for the commercial production of recombinant proteins. Until now, the expression of heterologous genes in chloroplasts is the only approach that has led to protein accumulation at economically viable levels [8,13,14]. However, nuclear expression provides the recombinant proteins with desirable characteristics, which cannot be acquired if they are expressed in the chloroplasts, such as post-translational modifications or targeting of the protein products to different organelles [15,16].

The nuclear transformation of microalgae takes place mainly by random integration. This means that transgenes are randomly inserted in the genome, and consequently their expression level will largely depend on a series of factors such as the insertion site, the environment genome surrounding of the inserted transgene, the number of copies inserted or differences with the codon usage of the host [10]. Random insertion followed by an exhaustive phenotypic screening of a large number of clones continues being the most usual approach for the selection of transformants with good levels of expression and stability [17,18]. However, different imaginative solutions have been proposed to overcome these time-consuming screenings and to get high expression of the transgenes. Improvement of promoters and regulatory regions (HSP70A, RbcS2 intron) [19,20,21,22,23], inclusion of additional introns [24] or adaptation of the codon usage of the transgene to that of the host microalga [25,26,27] have shown to improve expression and stability of marker genes such as GFP and luciferase in *Chlamydomonas reinhardtii*. We have previously demonstrated that fusing the gene of interest without promoter to an antibiotic resistance gene can lead to high expression [28]. Neupert and coworkers described high accumulation of proteins encoded by nuclear transgenes (about 0.2% of total soluble proteins) by UV induced mutagenesis of the obtained transformants followed by screening to isolate defective mutants in transgene suppression mechanisms [29]. A similar strategy was proposed by Kong and coworkers, who studied expression of proteins in a mutant of *Chlamydomonas* affected in a methyltransferase involved in transcriptional gene silencing [30].

A further improvement has been achieved by including the self-cleaving 2A peptide derived from the foot-and-mouth disease virus (FMDV-2A) between the *GOI* and the selectable gene. This peptide is processed in the eukaryotic ribosome due to a ribosomal skip between Gly23 and Pro24 amino acids [31]. The use of this small peptide for the co-expression of several genes under the same promoter and then, thanks to its co-translational cleavage ability obtain the independent gene products, has been applied to a wide range of eukaryotic systems, such as mammalian cells [32], fungi [33] and plants [34]. This strategy has also been shown to work in microalgae [30,35]. Mayfield and coworkers described the fusion of the bleomycin resistance gene (*ShBLE*) with the xylanase gene of *Trichoderma reesei* [35] and several fluorescent marker genes [16] through the small self-cleaving 2A peptide and demonstrated the obtaining of high yields of the independent proteins from a common transcript [36]. However, the high mutagenic effect of bleomycin and related antibiotics [37,38] and the need of extending the number of selective agents available have led us to propose an alternative gene fusion strategy to improve transgenes expression in microalgae based on the *APHVIII* resistance gene. This gene encodes the enzyme aminoglycoside 3′-phosphotransferase from *Streptomyces rimosus* and confers resistance to the antibiotic paromomycin [39]. We have generated different multicistronic expression plasmids in which the polylinker region is fused through the short self-cleaving 2A peptide to both termini of the selective *APHVIII* gene and evaluated their efficiency for the genetic transformation of the microalgae *Chlamydomonas reinhardtii*. Moreover, we have demonstrated that screening of the transformants with increasing amounts of the antibiotic paromomycin provides a simple method for the selection of clones with the highest level of expression of *APHVIII* gene, and consequently of the gene of interest, among the obtained transformants.

## 2. Results and Discussion

### 2.1. Construction and Transformation Efficiency of Several Multicistronic APHVIII-based Fusion Plasmids

**Figure 1:**
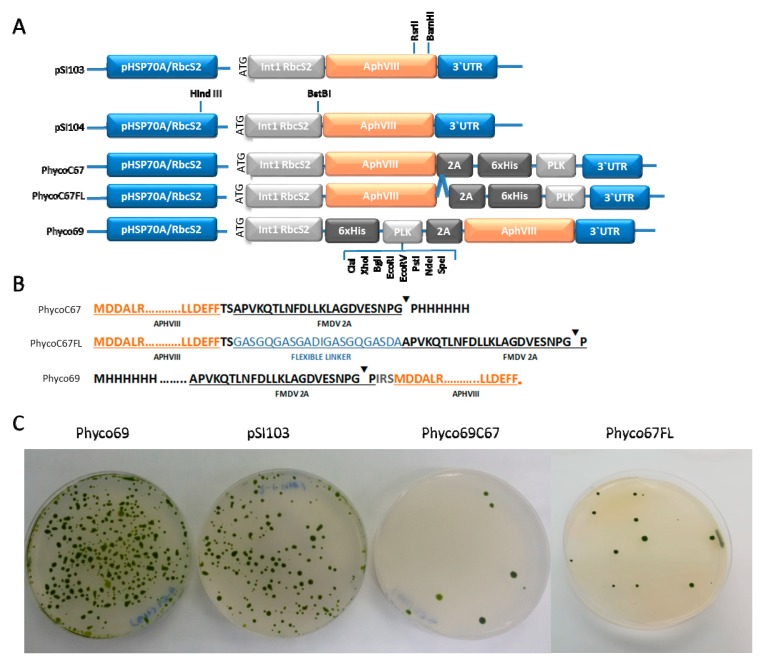
Schematic diagram of the main elements of the three new plasmids generated in this work in comparison with the control pSI103 plasmid (**A**) and of the translation products resulting from them (**B**). The blue line “**^**“ represents the flexible peptide sequence GASGQGASGADIGASGQGASDA. “▼“ denotes the hydrolysis point in the self-cleaving peptide FMDV-2A. Representative examples of the nuclear transformation of *Chlamydomonas reinhardtii* with the three newly generated plasmids (Phyco69, PhycoC67, and PhycoC67FL) and the control pSI103 plasmid are also shown (**C**).

Three different plasmids containing the paromomycin resistance gene, *APHVIII*, fused to a polylinker region through the foot-and-mouth disease virus self-cleaving 2A peptide sequence (FMDV-2A) have been constructed. In the three plasmids the *APHVIII* gene is placed under the control of the heat shock protein 70A/ribulose-1,5-bisphosphate carboxylase/oxygenase small subunit 2 tandem chimeric promoter (HSP70A-RBCS2) and terminated by the 3′RBCS2 untranslated region [19]. In addition, in the three plasmids the first intron of the Rubisco small subunit, which has shown to increase transformation efficiencies, has been included after the promoters and immediately before the corresponding marker gene or polylinker region, as detailed in Figure 1A. Plasmid pSI103 described by Sizova was used as a control plasmid [40]. The first construct encodes for the APHVIII protein linked through its carboxyl end (C-end) to the FMDV-2A peptide, which is followed by the polylinker region. The resulting plasmid was called PhycoC67. In the second construct, the C-end of the *APHVIII* sequence was linked to the self-cleaving 2A peptide through a flexible peptide sequence (GASGQGASGADIGASGQGASDA), this plasmid was denoted as PhycoC67FL. In the third construct, the same elements used for plasmid PhycoC67 were placed in inverse order, so that the PLK region followed by the self-cleaving 2A peptide is fused to the amino-end (N-end) of the *APHVIII* gene, this last plasmid was named Phyco69. The detailed schematic diagrams of this construct are shown in Figure 1A, and the sequences of the proteins obtained from their expression are shown in Figure 1B.

The 2A peptide allows that the chimeric transcripts generated from these constructions are processed upon translation in the ribosome, generating independent gene products, the APHVIII, and the one corresponding to the gene inserted in the polylinker region. The ribosomal skip takes place between the two final C-terminal amino acids of the 2A peptide, glycine, and proline, and consequently an extra peptidic sequence will be added to the C-end of the APHVIII when it is situated upstream the 2A peptide, as it happens in plasmids PhycoC67 or PhycoC67FL. However, in the case of plasmid Phyco69, the *APHVIII* gene is located downstream the 2A peptide, and consequently, a single proline amino acid will be added to its N-terminal.

The model microalga *Chlamydomonas reinhardtii* was transformed by the glass beads agitation method with the three plasmids, PhycoC67, PhycoC67FL, and Phyco69, as described in Section 3.3 Materials and Methods. The efficiency of these transformations was compared with that obtained with the control plasmid pSI103 [40]. As shown in Figure 1C, the transformation efficiency of PhycoC67 was extremely low, showing that even small peptides such as the FMDV-2A self-cleaving peptide interfere with the functionality of the aminoglycoside 3’-phosphotransferase if these are bound to its carboxyl terminal end. Protein linkers imitate the linkers naturally occurring in multi-domain proteins and usually avoid misfolding or impaired bioactivity of the fusion proteins, however, the flexible peptide used in PhycoC67FL did not offer any appreciable advantage. By contrast, Phyco69 plasmid, in which the FMDV-2A is linked to the amino terminal of the APHVIII protein, provided the highest transformation efficiencies with around 420 transformants µg^−1^ of DNA. This is three-fold the mean transformation efficiency obtained with the control pSI103 plasmid designed by Sizova [39], which is about 130 transformants µg^−1^ of DNA (Table 1).

**Figure A1:**
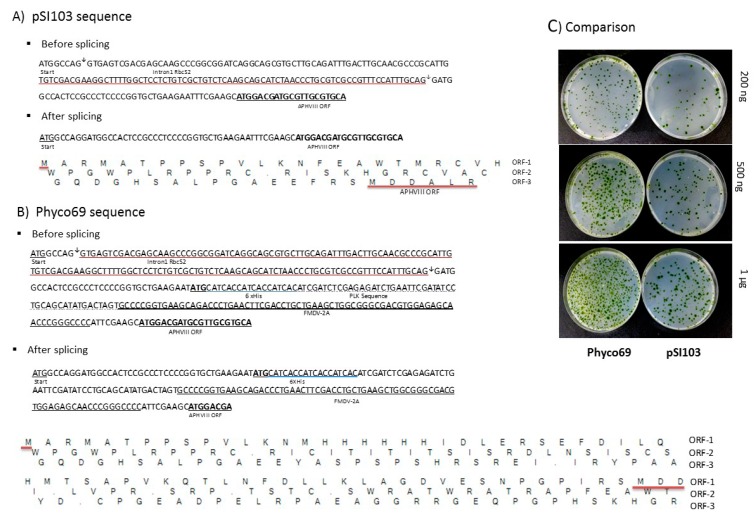
Detailed sequences flanking the start codon of the *APHVIII* gene before and after splicing of the first RbcS2 intron in the control pSI103 (**A**) and Phyco69 (**B**) plasmids and amino acidic sequence of the proteins generated in the three possible reading frames. A comparison of the efficiency of the transformation of *Chlamydomonas* with different quantities of each plasmid is also shown (**C**).

We concluded that the fusion of peptides through the amino-end does not affect the functionality of the aminoglycoside 3’-phosphotransferase, however, the fusion to the carboxyl-end of even small peptides such as the 2A peptide, interferes with its function. Although there is certain variability in the number of transformants obtained with these plasmids, we systematically obtained much higher transformation efficiencies with the plasmid Phyco69, around three times higher than with the control plasmid pSI103. After a careful analysis, we found that the increase in the transformation efficiency with the new Phyco69 plasmid is due to the fact that in Phyco69 the *APHVIII* gene is in the same reading frame than the initiation codon, which is immediately before the RbcS2 intron. Whereas in pSI103 the initiation codon does not match with the open reading frame of the APHVIII protein. Detailed sequences of the products obtained after the elimination of the RbcS2 intron in pSI103 and Phyco69 are shown in Figure A1. In pSI103 a mix of transcripts with different reading frames must be produced and the selective pressure of the antibiotic makes possible that those transformants in which the correct APHVIII protein is synthesized survive. The detailed map of the Phyco69 plasmid, the one chosen for further studies, can be found on PhycoGenetics website (http://phycogenetics.com/products/).

This plasmid allows higher transformation efficiencies than other plasmids with the same promoter. In addition, selection based on the antibiotic paromomycin is fast and clean, without spontaneous resistant clones or mutagenic effects [10]. The placement of the polyclonal region upstream the aminoglycoside 3’-phosphotransferase solves the problems generated when additional peptide sequences are fused to the carboxyl-end of this protein. In this case, the desired genes have to be deprived of their stop codon before being cloned in the Phyco69 plasmid to allow the transcription of the whole transcription unit. However, this apparent disadvantage ensures that all the transformants surviving in the presence of paromomycin are also expressing the *GOI*, which is placed upstream the *APHVIII*. If the *GOI* was placed downstream the resistance gene, some of the transformants could contain truncated transcripts in which the *GOI* is not transcribed. Since nuclear DNA insertion in microalgae is usually accompanied of deletions or truncations both in the genomic and insert DNA [41], ensuring the expression of the target gene by placing it upstream the selectable marker can be very useful.

Generation of fusion recombinant proteins or even the addition of short protein sequences to the ends of a protein can interfere with its structure and function. The possible influence of the remnants from the short 2A peptide in each protein of interest has to be investigated for each case. In the case of the APHVIII protein, we have demonstrated that the addition of even a few amino acidic units to its carboxylic-end affects its functionality. The few cases found in the literature, which describe the fusion of APHVIII to other proteins, involve its link through the amino end [18,40]. The structure of the APHVIII protein has been determined by X-ray crystallography [42] and is available in the protein data bank (PDB 4H05). APHVIII is a dimeric protein, each monomer consists of two lobes, the N-terminal domain with a β-strand motif of five anti-parallel sheets and two α-helices, and the catalytic C-domain with six α-helices. The three-dimensional structure of the APHVIII protein confirmed the conclusions previously established on the basis of sequence comparisons and point mutations, that pointed the final amino acid Phe267, as one of the conserved residues essential for the direct interaction with the aminoglycoside substrate [40]. This can explain why plasmid PhycoC67, in which the polylinker region is downstream the *APHVIII* sequence fused to its carboxylic-end, shows such a low transformation efficiency.

### 2.2. Phyco69 Enables the Selection of Transformants with High Expression Levels of the Gene of Interest

**Figure 2:**
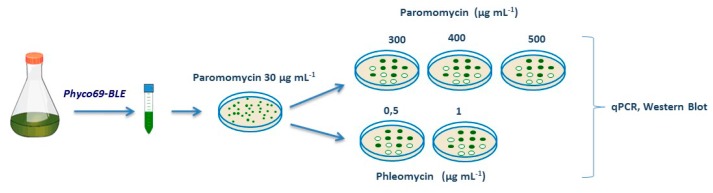
Schematic diagram of the selective strategy used to choose *Chlamydomonas* transformants with high levels of expression of genes *APHVIII* and *ShBLE*.

Our hypothesis is that by subjecting the transformants obtained with the plasmid Phyco69 to screening with increasing concentrations of the antibiotic paromomycin, it will be possible to select those clones which accumulate high concentrations of the aminoglycoside 3′-phosphotransferase protein and consequently of our protein of interest. Furthermore, both the protein of interest and the protein conferring resistance to the antibiotic are processed from the same multicistronic transcript, and if the transformants are maintained under selective conditions, the synthesis of the protein of interest is guaranteed to enhance in this way the stability of the transgenes. To validate this hypothesis, we cloned the antibiotic-resistance *ShBLE* gene, which confers resistance to antibiotics of the bleomycin family in the polylinker region of Phyco69 plasmid. The *ShBLE* gene with the first intron of RbcS2, which has shown to increase stability and transformation efficiency of the *ShBLE* gene [19,43], was PCR-amplified and inserted in the polylinker of Phyco69. The resulting plasmid, Phyco69BLE was used for the genetic transformation of *Chlamydomonas*. The layout of the experiment is depicted in Figure 2. Initial transformants selected in the presence of 30 µg·mL^−1^ of paromomycin appeared after four or five days and were screened with increasing concentrations of paromomycin (300, 400, 500 µg·mL^−1^) and phleomycin (0.5, 1 µg mL^−1^). One hundred transformants were cultured in 2 mL of liquid TAP medium with paromomycin 15 µg mL^−1^ during 48 h. The cellular density of all the cultures was then adjusted to the same value, and a drop of each transformant culture was symmetrically spotted in an ordered array on TAP agar plates supplemented with 300, 400 or 500 µg·mL^−1^ of paromomycin or with 0.5 or 1 µg mL^−1^ of phleomycin, where they were grown for several days. All experiments were carried out in triplicate.

**Figure A2:**
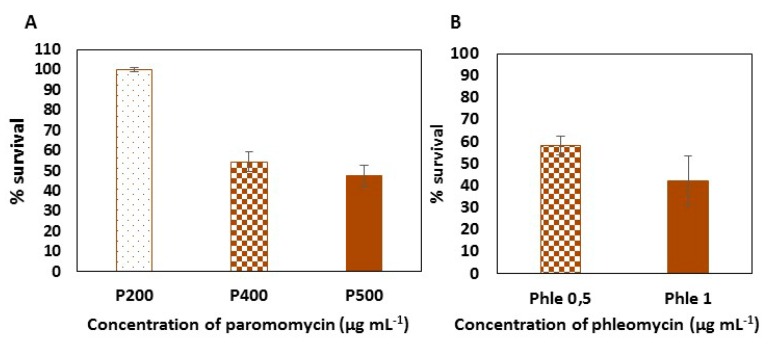
Average survival *Chlamydomonas* clones transformed with the plasmid Phyco69BLE and cultured with increasing concentrations of paromomycin (**A**) or phleomycin (**B**). Values are the average of at least three replicates and bars represent the standard deviation.

The average survival rate after transference to 500 µg mL^−1^ of paromomycin was 55%, very similar to the average survival rate in the presence of 1 µg mL^−1^ phleomycin, which was about 57% (Figure A2). We observed that practically all the transformed clones able to grow in the presence of high concentrations of paromomycin (500 µg mL^−1^) are also able to grow vigorously with phleomycin (1 µg mL^−1^). This indicates that these clones have high levels of expression of both *APHVIII* and *ShBLE* genes because it has been previously reported that high levels of the bleomycin binding protein, encoded by the *ShBLE* gene, are necessary to show a phleomycin resistance phenotype [35,43].

**Figure 3:**
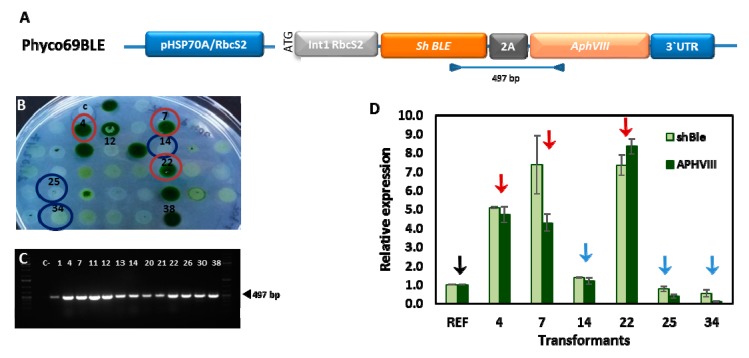
Relative expression of genes *ShBLE* and *APHVIII* in a series of *Chlamydomonas* transformants resistant to paromomycin. *Chlamydomonas* was transformed with the plasmid Phyco69BLE (**A**), and a selection of clones resistant to paromomycin and phleomycin (**B**) were analyzed by PCR using genomic DNA as target to test correct insertion of the *BLE-2A-APHVIII* cassette (**C**), and by real-time PCR (**D**) to test the relative expression level of the genes *ShBLE* (■) and *APHVIII* (■). Red arrows indicate the clones which show an important increase (>5 times) in the expression levels of *APHVIII* and *ShBLE* in relation with the reference transformed clone (**↓**), and correspond to the clones circled with a red line in panel B. Blue arrows denote the clones with a small difference in the expression of these genes in relation with the reference transformed clone and correspond to the clones circled with a blue line in panel (**B**). The expression of all genes was normalized to the ubiquitin ligase (*UBQL*) house-keeping gene and presented as fold-change relative to the transcript level of the reference transformed clone, which was chosen among those transformants which show low expression levels. Values are the average of three replicates, and bars indicate standard deviation.

To confirm this, we studied the expression levels of *APHVIII* and *ShBLE* in a series of transformed clones obtained with the Phyco69BLE plasmid (Figure 3A). The correct simultaneous insertion of both, *APHVIII* and *ShBLE* genes, in the genome of the selected clones, was checked by PCR with specific primers that anneal at the end of the *ShBLE* gene and at the beginning of the *APHVIII* gene. All the clones checked had incorporated both genes correctly in their genomes (Figure 3C).

On the one hand, we studied three PAR^R^, BLE^R^-transformants that could grow in 30 µg mL^−1^ paromomycin but were not able to survive neither in paromomycin 500 µg mL^−1^ nor in phleomycin 1 µg mL^−1^ (#14, #25, #34). On the other hand, we selected three transformants able to grow vigorously both in paromomycin 500 µg mL^−1^ and in phleomycin 1 µg mL^−1^ (#4, #7, #22). The transcripts levels corresponding to both genes were determined by real-time PCR using specific primers for each gene and the ubiquitin ligase gene (*UBQL*) as housekeeping gene (Figure 3D).

As shown in Figure 3D, the *APHVIII* and *ShBLE* transcript levels in the transformants which tolerate high concentrations of paromomycin (500 µg mL^−1^) are between five and eight times the transcript levels found in the transformants isolated in 30 µg mL^−1^ paromomycin but unable to survive at higher concentrations of paromomycin (Figure 3B). This confirms that the selective pressure with paromomycin provides a fast method for the selection of the transformants with the highest levels of expression of the desired gene.

The self-cleaving peptide FMDV-2A has been successfully used in several eukaryotic systems [34]. Mayfield and coworkers have demonstrated in the microalga *Chlamydomonas reinhardtii*, that the FMDV-2A approach can be successfully used to fuse the desired gene to the *ShBLE* gene, which provides resistance to antibiotics of the bleomycin family [36]. The mechanism of action of BLE, which relies on antibiotic sequestration rather than enzymatic inactivation, ensures that the transformants surviving in these antibiotics have high levels of expression [43]. However, it is desirable to extend the palette of selectable markers available to be used with the FMDV-2A system because the selection of some microalgae must be done with other antibiotics to which they are more susceptible. In addition, bleomycin is highly mutagenic [37,44], and this is an undesirable effect, especially when an unequivocal relation between the obtained phenotype and the expressed gene must be established.

### 2.3. Phyco69 Enables the Simultaneous Expression of Two Genes, Which are Efficiently Processed Generating Independent Proteins

**Figure 4:**
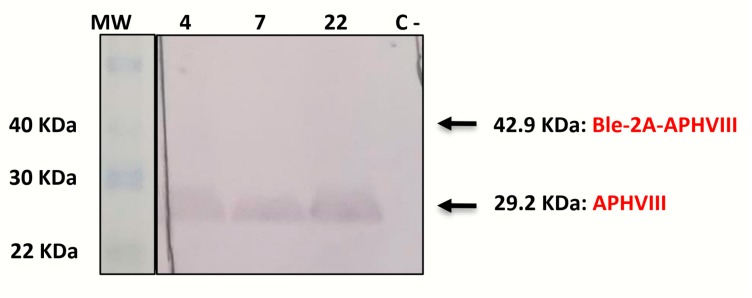
Immunoblot analysis showing the APHVIII protein product from *C. reinhardtii* cells transformed with the plasmid Phyco69BLE. Total soluble cell protein extracts (60 μg) of three *Chlamydomonas* transformants (#4, #7, #22) and one control untransformed *Chlamydomonas* culture (C-) were fractionated with SDS-PAGE, transferred to a PVDF membrane and probed with rabbit anti-APHVIII polyclonal antibodies and alkaline phosphatase-conjugated goat anti-rabbit IgG, as indicated in materials and methods. MW lane: Protein molecular weight markers.

**Figure A3:**
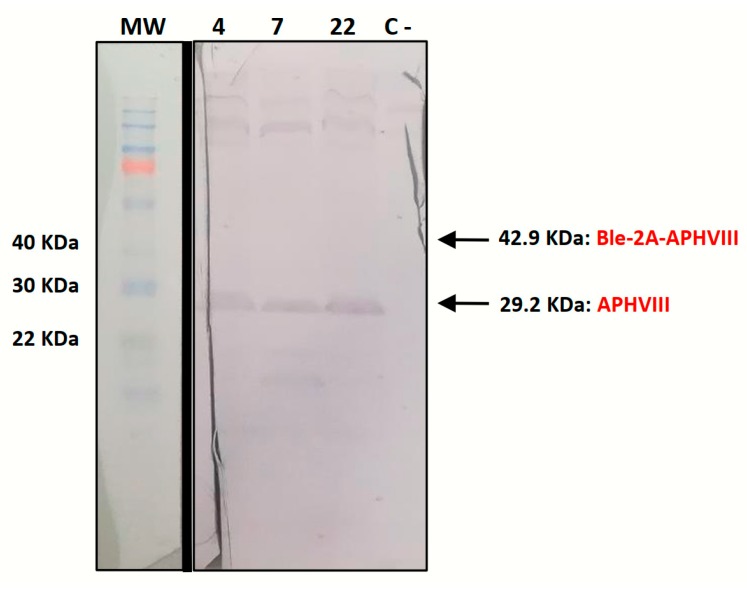
Inmunoblot analysis showing the APHVIII protein in the full-length blot. Total protein extracts (60 μg) were fractionated with SDS-PAGE, transferred to a PVDF membrane and probed with rabbit anti-APHVIII polyclonal antibodies and alkaline phosphatase-conjugated goat anti-rabbit IgG, as indicated in 3.4 materials and methods. MW, contains molecular weight markers; #4, #7 and #22 corresponding to three *Chlamydomonas* transformants and C-, one control untransformed *Chlamydomonas* culture.

Although the utility of the 2A peptide to generate independent mature proteins from a common RNA transcript has been well established, the cleavage efficiency of the 2A sequences can be influenced by the C-terminal of the protein upstream the 2A. In the present work, immunodetection studies using anti-APHVIII antibodies on the transformants obtained with Phyco69BLE plasmid showed a 29.2 KDa-band, which corresponds to the size of the APHVIII protein, while no band is detected at 42.9 KDa, the size expected for the fusion Ble-2A-APHVIII protein product (Figure 4, Figure A3).

This demonstrates that the multicistronic transcript is correctly processed generating independent proteins and no detectable fusion product. The 2A system has previously shown to improve expression of the squalene synthase from *Botryococcus braunii* [30] and several selectable marker proteins in *Chlamydomonas* [35,45]. In all these cases, the efficient generation of two independent protein products through the 2A peptide has been reported. Furthermore, this auto-cleaving peptide has allowed the successful expression of four independent proteins from a single vector in *Chlamydomonas* [18] and up to six different genes in human cell lines [46]. Other viral sequences similar to the foot-and-mouth disease 2A have been reported to undergo similar translational self-cleaving with different efficiencies [47]. Moreover, some authors have even reported the efficient expression of two genes from a single bicistronic mRNA by simply joining the two genes of interest by a short stretch of unstructured junction sequences, without the need of viral sequences [48]. Although further insights are necessary to clarify the precise mechanism of the 2A induced ribosome skip, multicistronic plasmids have important advantages over co-transformation approaches in which the selective marker and the gene of interest are in independent expression cassettes and usually result in low percentages of genome insertion of the desired gene.

## 3. Materials and Methods

### 3.1. Strains and Culture Conditions

*Chlamydomonas reinhardtii 704* strain (Cw15, Arg7^+^, mt^+^) was kindly donated by Dr. Roland Loppes (University of Liège, Sart Tilman, Belgium) [49], and cultured photomixotrophically in liquid or agar solidified Tris-acetate phosphate (TAP) medium under continuous white light irradiation (50 μE m^−2^ s^−1^ photosynthetically active radiation) at 25 ˚C in a culture chamber. The DH5α *Escherichia coli* strain, used for in vivo amplification of DNA, was cultured in an LB medium.

### 3.2. Plasmids Constructions

A synthetic DNA fragment (Genscript, Co, Piscataway, NJ, USA) flanked by the RsrII and the BamHI recognition sequences and consisting of 170 final nucleotides of the *APHVIII* gene without the stop codon, followed by the FMDV-2A, the 6 histidines encoding sequences and an artificial polylinker sequence, was cloned between the RsrII / BamHI sites of the plasmid pSI104 to obtain plasmid PhycoC67. Plasmid pSI104 [50] is a modification of plasmid pS103 [40] obtained from the *Chlamydomonas* Resource Center US (https://www.Chlamycollection.org). *APHVIII* encodes the enzyme aminoglycoside 3′-phosphotransferase *Streptomyces rimosus* and confers resistance to the antibiotic paromomycin. The FMDV-2A is a small self-cleaving peptide derived from the foot-and-mouth disease virus. Plasmid PhycoC67FL was prepared by including a synthetic DNA sequence that encodes the flexible peptide GASGQGASGADIGASGQGASDA between the *APHVIII* and the FMDV-2A sequences of plasmid PhycoC67. Plasmid Phyco69 available from PhycoGenetics SL., (Huelva, Spain) (http://www.phycogenetics.com) was constructed by including in front of the *APHVIII* gene of plasmid pSI104 a synthetic DNA fragment (Genscript, Co) consisting of the final nucleotides of the 5′ regulatory region of RbcS2, followed by the 6 histidines, the FMDV-2A encoding sequences, and an artificial polylinker. This DNA fragment was inserted between the HindIII and BstBI restriction sites of pSI104 (Figure 1). Phyco69BLE plasmid was obtained by inserting between the ClaI and SpeI restriction sites of plasmid Phyco69 a stop codon-less version of *Streptoalloteichus hindustanus BLE* gene. This version of the *ShBLE* gene contains the first intron of the *RBCS2* gene and confers resistance to bleomycin and related glycopeptide antibiotics, such as zeocin and phleomycin, upon binding to them.

### 3.3. Chlamydomonas Nuclear Transformation

The nuclear transformation of *C. reinhardtii* was carried out using the glass beads method of Kindle [51] with minor modifications. *Chlamydomonas* cultures were grown to a cell density of about 10^7^ cells mL^−1^, afterwards they were centrifugated, spinned down, and resuspended to get a final 100 fold concentrated cell suspension. 0.3 g of sterile glass beads (0.4–0.6 mm Ø) were added to 0.6 mL of concentrated cell suspension, 0.2 mL of 20% polyethylene glycol (MW8000), and the indicated quantity of the desired plasmid. This mixture was agitated during 10 s, and then cells were resuspended in 50 mL of fresh TAP medium and left in dim light overnight. After this incubation, cells were spread onto the selective solid medium carrying 30 µg mL^−1^ of paromomycin. Transformed colonies were visible after four or five days.

### 3.4. Real Time PCR

qPCR experiments were performed on the Mx3000P Multiplex Quantitative PCR System (Agilent Technology, La Jolla, CA, USA) using as template 1 μL of the cDNA, synthesized from total RNA according to the Invitrogen SuperScript II RNase-reverse transcriptase manual (Life Technologies Corporation, Carlsbad, CA, USA) and Brilliant SYBR^®^ Green QPCR Master Mix (Agilent Technologies, La Jolla, CA, USA). Cycling conditions were: 10 min at 95 °C for activation of the Hot start Taq polymerase and 40 cycles for the melting (30 s at 95 °C), annealing (30 s at 61 °C), and extension (30 s at 72 °C). Each qPCR measurement was carried out in triplicate using specific primers for either *ShBLE* or *APHVIII* (Table A1). The *UBC8* gene, encoding a ubiquitin ligase polypeptide (XM_001697453), whose expression was previously shown to be constitutive under the different conditions used [52,53] and was used as a housekeeping gene to normalize mRNA abundance. 2^−ΔΔCT^ approach was used to calculate fold change relative to the expression level of a reference clone, which was chosen among the transformants which showed the lowest values [54].

### 3.5. Western Blot

For Western blot analysis, proteins, separated by denaturing SDS-PAGE as previously described [55], were transferred from acrylamide gel onto a polyvinylidene difluoride membrane (PVDF) using the Mini Trans-Blot system (Bio-Rad, Hercules, CA, USA) according to the manufacturer’s instructions. PVDF membrane was washed with TBS and blocked overnight with 2.5% nonfat-dry milk containing 0.2% Tween 20. Immunodetection of APHVIIII in *C. reinhardtii* crude extracts was performed using anti-APHVIII protein antibodies, raised in rabbit immunized with the recombinant purified APHVIII protein (500 mg), as primary antibodies at 1:8000 dilution in TBS (0.5% nonfat-dry milk, 0.2% Tween 20) and alkaline phosphatase (AP)-conjugated goat-anti-rabbit IgG (SIGMA) at 1:20,000 dilution as secondary antibody.

## 4. Conclusions

Great efforts are being made to increase the level of expression of heterologous genes in the microalgal nucleus. The fusion of the genes of interest through the self-cleaving 2A peptide to a selectable marker is a good strategy for expression of foreign genes in microalgae, which to date has never been implemented using the versatile and efficient paromomycin resistant gene, *APHVIII*. Here we demonstrate the suitability of a plasmid containing this antibiotic-resistant marker to express independent proteins linked through the FMDV-2A peptide, as long as they are fused through its N-terminal. Paromomycin is a stable and reliable antibiotic which lacks the mutagenic effects of other antibiotics, such as bleomycin or related antibiotics, and allows quick isolation of transformants without spontaneous resistance. Plasmid Phyco69, designed to contain any gene of interest linked through the 2A fragment upstream of the *APHVIII* gene has been validated using the *ShBLE*, which encodes the bleomycin resistant protein as a gene of interest. Immunodetection studies showed that the multicistronic transcript generated from Phyco69 plasmid is correctly processed, generating independent gene products. Phenotypic studies and RT-PCR analysis have shown that screening the transformants with increasing amounts of the antibiotic paromomycin provides a simple method for the selection of clones with the highest level of expression of *APHVIII* gene, and consequently of the gene of interest. This plasmid can be used to express different exogenous proteins of interest or overexpress homologous proteins in this and other related microalgae.

## Figures and Tables

**Table 1:** Transformation efficiency of *Chlamydomonas reinhardtii* with increasing quantities of the indicated plasmids. All values are the average of between three and five repetitions ± SD. The mean number of transformants µg^−1^ of DNA is also indicated.

Plasmid	200 ng	500 ng	1 µg	2 µg	Transformants µg^−1^ DNA
Phyco69	98 ± 22	215 ± 38	380 ± 58	820 ± 70	427
pSI103	28 ± 12	60 ± 21	140 ± 32	250 ± 45	131
PhycoC67	0	5 ± 2	7 ± 4	18 ± 6	6.5
PhycoC67FL	0		8 ± 4	12 ± 7	3.5

## Appendix A

**Table A1:** Oligonucleotides used in this work for quantitative RT-PCR experiments.

Target Gene	Primer Name	Sequence
*BLE*	qBLE-F	CGACTTCGCCGGTGTGGTC
	qBLE-R	CACGAAGTGCACGCAGTTGC
*APHVIII*	q*APHVIII*F	GAGGATCTGGACGAGGAGCGGAA
	q*APHVIII*R	CCCTCAGAAGAACTCGTCCAACAGC
*UBC8*	qUBC8	GTACAGCGGCGGCTAGAGGCAC
	qUBC8	AGCGTCAGCGGCGGTTGCAGGTATCT

*BLE*, bleomycin resistance protein of *Streptoalloteichus hindustanus* (1BYL-A); *APHVIII*, aminoglycoside 3′-phosphotransferase from *Streptomyces rimosus* (NG_047446.1); *UBC8*, Ubiquitin protein ligase from *Chlamydomonas reinhardtii* (XM_001697453.1).

## References

[1] Leu S., Boussiba S. (2014). Advances in the Production of High-Value Products by Microalgae. Ind. Biotechnol..

[2] Benemann J. (2013). Microalgae for Biofuels and Animal Feeds. Energies.

[3] Varela J.C., Pereira H., Vila M., León R. (2015). Production of carotenoids by microalgae: Achievements and challenges. Photosynth. Res..

[4] Gangl D., Zedler J.A.Z., Rajakumar P.D., Martinez E.M.R., Riseley A., Włodarczyk A., Purton S., Sakuragi Y., Howe C.J., Jensen P.E. (2015). Biotechnological exploitation of microalgae. J. Exp. Bot..

[5] Valverde F., Romero-Campero F.J., León R., Guerrero M.G., Serrano A. (2016). New challenges in microalgae biotechnology. Eur. J. Protistol..

[6] Gifuni I., Pollio A., Safi C., Marzocchella A., Olivieri G. (2019). Current Bottlenecks and Challenges of the Microalgal Biorefinery. Trends Biotechnol..

[7] Sharon-Gojman R., Maimon E., Leu S., Zarka A., Boussiba S. (2015). Advanced methods for genetic engineering of Haematococcus pluvialis (Chlorophyceae, Volvocales). Algal Res..

[8] Doron L., Segal N., Shapira M. (2016). Transgene Expression in Microalgae-From Tools to Applications. Front. Plant Sci..

[9] Jeon S., Lim J.-M., Lee H.-G., Shin S.-E., Kang N.K., Park Y.-I., Oh H.-M., Jeong W.-J., Jeong B., Chang Y.K. (2017). Current status and perspectives of genome editing technology for microalgae. Biotechnol. Biofuels.

[10] León-Bañares R., González-Ballester D., Galván A., Fernández E. (2004). Transgenic microalgae as green cell-factories. Trends Biotechnol..

[11] Vazquez-Villegas P., Torres-Acosta M.A., Garcia-Echauri S.A., Aguilar-Yanez J.M., Rito-Palomares M., Ruiz-Ruiz F. (2018). Genetic manipulation of microalgae for the production of bioproducts. Front. Biosci..

[12] Charoonnart P., Purton S., Saksmerprome V. (2018). Applications of Microalgal Biotechnology for Disease Control in Aquaculture. Biology.

[13] Potvin G., Zhang Z. (2010). Strategies for high-level recombinant protein expression in transgenic microalgae: A review. Biotechnol. Adv..

[14] Purton S., Szaub J.B., Wannathong T., Young R., Economou C.K. (2013). Genetic engineering of algal chloroplasts: Progress and prospects. Russ. J. Plant Physiol..

[15] Surzycki R., Greenham K., Kitayama K., Dibal F., Wagner R., Rochaix J.-D., Ajam T., Surzycki S. (2009). Factors effecting expression of vaccines in microalgae. Biologicals.

[16] Rasala B.A., Barrera D.J., Ng J., Plucinak T.M., Rosenberg J.N., Weeks D.P., Oyler G.A., Peterson T.C., Haerizadeh F., Mayfield S.P. (2013). Expanding the spectral palette of fluorescent proteins for the green microalga *Chlamydomonas reinhardtii*. Plant J..

[17] Hallmann A. (2007). Algal Transgenics and Biotechnology. Transgenic Plant J..

[18] Lauersen K.J., Kruse O., Mussgnug J.H. (2015). Targeted expression of nuclear transgenes in *Chlamydomonas reinhardtii* with a versatile, modular vector toolkit. Appl. Microbiol. Biotechnol..

[19] Schroda M., Beck C.F., Vallon O. (2002). Sequence elements within an HSP70 promoter counteract transcriptional transgene silencing in *Chlamydomonas*. Plant J..

[20] Fischer N., Rochaix J.D. (2001). The flanking regions of PsaD drive efficient gene expression in the nucleus of the green alga *Chlamydomonas reinhardtii*. Mol. Genet. Genom..

[21] Wu J., Hu Z., Wang C., Li S., Lei A. (2008). Efficient expression of green fluorescent protein (GFP) mediated by a chimeric promoter in *Chlamydomonas reinhardtii*. Chin. J. Oceanol. Limnol..

[22] Scranton M.A., Ostrand J.T., Georgianna D.R., Lofgren S.M., Li D., Ellis R.C., Carruthers D.N., Dräger A., Masica D.L., Mayfield S.P. (2016). Synthetic promoters capable of driving robust nuclear gene expression in the green alga *Chlamydomonas reinhardtii*. Algal Res..

[23] Crozet P., Navarro F.J., Willmund F., Mehrshahi P., Bakowski K., Lauersen K.J., Pérez-Pérez M.-E., Auroy P., Gorchs Rovira A., Sauret-Gueto S. (2018). Birth of a Photosynthetic Chassis: A MoClo Toolkit Enabling Synthetic Biology in the Microalga *Chlamydomonas reinhardtii*. ACS Synth. Biol..

[24] Baier T., Kros D., Feiner R.C., Lauersen K.J., Müller K.M., Kruse O. (2018). Engineered Fusion Proteins for Efficient Protein Secretion and Purification of a Human Growth Factor from the Green Microalga *Chlamydomonas reinhardtii*. ACS Synth. Biol..

[25] Fuhrmann M., Oertel W., Hegemann P. (1999). A synthetic gene coding for the green fluorescent protein (GFP) is a versatile reporter in *Chlamydomonas reinhardtii*. Plant J..

[26] Fuhrmann M. (2004). Production of antigens in *Chlamydomonas reinhardtii*: Green microalgae as a novel source of recombinant proteins. Methods Mol. Med..

[27] Shao N., Bock R. (2008). A codon-optimized luciferase from Gaussia princeps facilitates the in vivo monitoring of gene expression in the model alga *Chlamydomonas reinhardtii*. Curr. Genet..

[28] Díaz-Santos E., Vila M., Vigara J., León R. (2016). A new approach to express transgenes in microalgae and its use to increase the flocculation ability of *Chlamydomonas reinhardtii*. J. Appl. Phycol..

[29] Neupert J., Karcher D., Bock R. (2009). Generation of *Chlamydomonas* strains that efficiently express nuclear transgenes. Plant J..

[30] Kong F., Yamasaki T., Kurniasih S.D., Hou L., Li X., Ivanova N., Okada S., Ohama T. (2015). Robust expression of heterologous genes by selection marker fusion system in improved *Chlamydomonas* strains. J. Biosci. Bioeng..

[31] Szymczak A.L., Workman C.J., Wang Y., Vignali K.M., Dilioglou S., Vanin E.F., Vignali D.A.A. (2004). Correction of multi-gene deficiency in vivo using a single “self-cleaving” 2A peptide-based retroviral vector. Nat. Biotechnol..

[32] Minskaia E., Nicholson J., Ryan M.D. (2013). Optimisation of the foot-and-mouth disease virus 2A co-expression system for biomedical applications. BMC Biotechnol..

[33] Subramanian V., Schuster L.A., Moore K.T., Taylor L.E., Baker J.O., Vander Wall T.A., Linger J.G., Himmel M.E., Decker S.R. (2017). A versatile 2A peptide-based bicistronic protein expressing platform for the industrial cellulase producing fungus, Trichoderma reesei. Biotechnol. Biofuels.

[34] Luke G.A., De Felipe P., Cowton V.M., Hughes L.E., Halpin C., Ryan M.D. (2006). Self-processing polyproteins: A strategy for co-expression of multiple proteins in plants. Biotechnol. Genet. Eng. Rev..

[35] Rasala B.A., Lee P.A., Shen Z., Briggs S.P., Mendez M., Mayfield S.P. (2012). Robust expression and secretion of Xylanase1 in *Chlamydomonas reinhardtii* by fusion to a selection gene and processing with the FMDV 2A peptide. PLoS ONE.

[36] Rasala B.A., Chao S.-S., Pier M., Barrera D.J., Mayfield S.P. (2014). Enhanced Genetic Tools for Engineering Multigene Traits into Green Algae. PLoS ONE.

[37] Gonzalez-Ballester D., de Montaigu A., Higuera J.J., Galvan A., Fernandez E. (2005). Functional genomics of the regulation of the nitrate assimilation pathway in *Chlamydomonas*. Plant Physiol..

[38] Kong F., Yamaoka Y., Ohama T., Lee Y., Li-Beisson Y. (2019). Molecular Genetic Tools and Emerging Synthetic Biology Strategies to Increase Cellular Oil Content in *Chlamydomonas reinhardtii*. Plant Cell Physiol..

[39] Sizova I.A., Lapina T.V., Frolova O.N., Alexandrova N.N., Akopiants K.E., Danilenko V.N. (1996). Stable nuclear transformation of *Chlamydomonas reinhardtii* with a Streptomyces rimosus gene as the selective marker. Gene.

[40] Sizova I., Fuhrmann M., Hegemann P. (2001). A Streptomyces rimosus aphVIII gene coding for a new type phosphotransferase provides stable antibiotic resistance to *Chlamydomonas reinhardtii*. Gene.

[41] Jinkerson R.E., Jonikas M.C. (2015). Molecular techniques to interrogate and edit the *Chlamydomonas* nuclear genome. Plant J..

[42] Boyko K.M., Gorbacheva M.A., Korzhenevskiy D.A., Alekseeva M.G., Mavletova D.A., Zakharevich N.V., Elizarov S.M., Rudakova N.N., Danilenko V.N., Popov V.O. (2016). Structural characterization of the novel aminoglycoside phosphotransferase AphVIII from Streptomyces rimosus with enzymatic activity modulated by phosphorylation. Biochem. Biophys. Res. Commun..

[43] Fordham-Skelton A.P., Chilley P., Lumbreras V., Reignoux S., Fenton T.R., Dahm C.C., Pages M., Gatehouse J.A. (2002). A novel higher plant protein tyrosine phosphatase interacts with SNF1-related protein kinases via a KIS (kinase interaction sequence) domain. Plant J..

[44] Hoffmann G.R., Ronan M.V., Sylvia K.E., Tartaglione J.P. (2009). Enhancement of the recombinagenic and mutagenic activities of bleomycin in yeast by intercalation of acridine compounds into DNA. Mutagenesis.

[45] Jiang W., Brueggeman A.J., Horken K.M., Plucinak T.M., Weeks D.P. (2014). Successful transient expression of Cas9 and single guide RNA genes in *Chlamydomonas reinhardtii*. Eukaryot. Cell.

[46] Xu T., Ripp S., Sayler G.S., Close D.M. (2014). Expression of a Humanized Viral 2A-Mediated lux Operon Efficiently Generates Autonomous Bioluminescence in Human Cells. PLoS ONE.

[47] Sun H., Zhou N., Wang H., Huang D., Lang Z. (2017). Processing and targeting of proteins derived from polyprotein with 2A and LP4/2A as peptide linkers in a maize expression system. PLoS ONE.

[48] Onishi M., Pringle J.R. (2016). Robust transgene expression from bicistronic mRNA in the green alga *Chlamydomonas reinhardtii*. G3.

[49] Loppes R., Radoux M., Ohresser M.C., Matagne R.F. (1999). Transcriptional regulation of the Nia1 gene encoding nitrate reductase in *Chlamydomonas reinhardtii*: Effects of various environmental factors on the expression of a reporter gene under the control of the Nia1 promoter. Plant Mol. Biol..

[50] León R., Couso I., Fernández E. (2007). Metabolic engineering of ketocarotenoids biosynthesis in the unicelullar microalga *Chlamydomonas reinhardtii*. J. Biotechnol..

[51] Kindle K.L. (1990). High-frequency nuclear transformation of *Chlamydomonas reinhardtii*. Proc. Natl. Acad. Sci. USA.

[52] Gonzalez-Ballester D., Pootakham W., Mus F., Yang W., Catalanotti C., Magneschi L., de Montaigu A., Higuera J.J., Prior M., Galvan A. (2011). Reverse genetics in *Chlamydomonas*: A platform for isolating insertional mutants. Plant Methods.

[53] Vila M., Couso I., León R. (2008). Carotenoid content in mutants of the chlorophyte *Chlamydomonas reinhardtii* with low expression levels of phytoene desaturase. Process. Biochem..

[54] Pfaffl M.W. (2001). A new mathematical model for relative quantification in real-time RT-PCR. Nucleic Acids Res..

[55] Díaz-Santos E., de la Vega M., Vila M., Vigara J., León R. (2013). Efficiency of different heterologous promoters in the unicellular microalga *Chlamydomonas reinhardtii*. Biotechnol. Prog..

